# Delayed Post-pancreaticoduodenectomy Chylous Ascites: A Case of Cisterna Chyli Leak Managed With Lymphatic Embolization

**DOI:** 10.7759/cureus.107670

**Published:** 2026-04-24

**Authors:** Justin Peterson, Tony Kamel, Nathaniel Meyer, Adrian Mayo, Michael F Ayoub

**Affiliations:** 1 Department of Medicine, University of California Los Angeles David Geffen School of Medicine, Los Angeles, USA

**Keywords:** ascites, chyloperitoneum, chylous ascites, interventional radiology-guided embolization, whipple procedure

## Abstract

Chyloperitoneum is a rare cause of ascites that results from the leakage of lipid-rich lymph into the abdominal cavity. Numerous etiologies can cause chylous ascites, with the most common causes being malignancy, cirrhosis, infection, and post-procedural lymphatic duct injury in adults. We describe a case of delayed chylous ascites formation after a pancreaticoduodenectomy (Whipple) procedure with lymphocele formation and slow leakage into the peritoneal cavity. Prompt recognition and diagnosis are paramount given the high mortality rate, and the management of this condition includes treating the underlying cause, symptom management, nutritional modification, and, in select cases, surgical interventions such as embolization.

## Introduction

Chyloperitoneum is a rare cause of ascites and occurs through one of three pathophysiologic mechanisms: the obstruction of lymph flow due to malignancy; an injury to major abdominal lymphatic channels, including the cisterna chyli, causing the leakage of lymph into the abdominal cavity; or the exudation of lymph through dilated retroperitoneal vessel walls with fistulous leak into the peritoneal cavity [1,2]. It is most frequently caused by malignancy, cirrhosis, and lymphatic duct injury after abdominal surgery in adults [3]. Chyle plays an essential role in immune function and nutritional homeostasis, due to its composition of lymphocytes, proteins, triglycerides, and fat-soluble vitamins [4]. About 2-4 L of chyle flows through the lymphatic system daily, and a chylous leak can lead to immunocompromise and malnutrition. Infections, lymphopenia, hypoalbuminemia, vitamin deficiencies, hypovolemia, and electrolyte derangements, including hyponatremia and hypocalcemia, are frequently encountered.

In patients presenting with chylous ascites due to lymphatic duct injury during surgery, the onset of ascites and abdominal distension may be rapid, but delayed presentations, as seen in this case, can be due to lymphocele formation, slow leakage, or other various pathophysiologic mechanisms [4]. The diagnosis may be obtained through the fluid analysis of the ascites, which is marked by elevated triglycerides of levels above 200 mg/dL, though others have proposed a threshold as low as 110 mg/dL [5,6]. The classic milky-white appearance the fluid may demonstrate is consistent with the diagnosis, though this color is neither diagnostic nor necessary for the diagnosis [7]. Imaging may also be helpful, including computed tomography (CT) scan to evaluate for masses or enlarged lymph nodes, magnetic resonance (MR) lymphography, lymphangiography, and lymphoscintigraphy, which can detect structural abnormalities, fistulas, and/or the leakage of lymphatic fluid [8]. Management involves the diagnostic and therapeutic removal of ascitic fluid, with evaluation for secondary causes and/or alternative diagnoses, including cirrhosis, infections, and malignancies. The initiation of a high-protein, low-fat diet with medium-chain triglycerides and the supplementation of fat-soluble vitamins is advised [9]. Medical management is used in many cases to allow for the healing of a fistulous tract or leak, while other cases may require or benefit from the procedural intervention of the underlying pathology, such as the embolization of a lymphatic leak, tumor debulking/removal, transjugular intrahepatic portosystemic shunt (TIPS) in cirrhosis, or treating an active infection [9]. As this condition represents a relative minority of cases of ascites and does not always present with a rapid onset of ascites or with the classic “milky-white” ascitic fluid appearance, a high degree of clinical suspicion is important for recognition and diagnosis. Here, we report a case of progressive chylous ascites with delayed development after pancreaticoduodenectomy, diagnosed through elevated triglycerides in the ascitic fluid analysis and characteristic findings on lymphangiography, with definitive management by cisterna chyli embolization.

## Case presentation

A 59-year-old man with a significant past medical history of an ampullary neuroendocrine carcinoma status post (s/p) Whipple, end-stage renal disease (ESRD) secondary to adult polycystic kidney disease on hemodialysis, left lower extremity deep vein thrombosis, hypertension, and the avascular necrosis of the right hip presented with several days of weakness, poor oral intake, and progressive abdominal distension. The patient had been diagnosed with an ampullary neuroendocrine carcinoma three months prior to admission and underwent a robotic-assisted laparoscopic pancreaticoduodenectomy resection two months prior to admission, with resected tissue pathology subsequently revealing negative gross margins for malignancy. He had resultant pancreatic insufficiency that was treated with supplemental oral pancreatic enzymes. Given the patient’s progressive weakness and abdominal distension, he reported being unable to undergo any intermittent hemodialysis sessions for one week prior to admission.

On admission, the patient was afebrile with a heart rate of 80 beats per minute, blood pressure of 144/76 mmHg, respiratory rate of 14 breaths per minute, and oxygen saturation of 99% on ambient air. His physical examination was notable for being alert and oriented to person, place, and time. His lungs were clear to auscultation bilaterally, and his abdomen was distended and diffusely tender to palpation without rebound or guarding. Spider angiomas and palmar erythema were not present on dermatologic inspection. His initial laboratory workup is summarized in Table 1. The patient was admitted to the intensive care unit and underwent several sessions of intermittent hemodialysis with improvement in his electrolyte abnormalities.

**Table 1:** Initial Laboratory Values

Laboratory Test	Result	Reference Range
White Blood Cell Count	10.76 × 10E3/μL	4.16-9.95 × 10E3/μL
Hemoglobin	8.8 g/dL	11.6-15.2 g/dL
Platelets	400 × 10E3/μL	143-398 × 10E3/μL
Sodium	140 mmol/L	135-146 mmol/L
Potassium	7.4 mmol/L	3.6-5.3 mmol/L
Bicarbonate	14 mmol/L	20-30 mmol/L
Blood Urea Nitrogen (BUN)	136 mg/dL	7-22 mg/dL
Creatinine	20.11 mg/dL	0.6-1.3 mg/dL
Aspartate Aminotransferase (AST)	26 U/L	13-62 U/L
Alanine Aminotransferase (ALT)	24 U/L	8-70 U/L
Alkaline Phosphatase	121 U/L	37-113 U/L
Total Bilirubin	0.5 mg/dL	0.1-1.2 mg/dL
Albumin	3.3 g/dL	3.9-5.0 g/dL
Total Protein	6.5 g/dL	6.1-8.2 g/dL
Calcium	9.8 mg/dL	8.6-10.4 mg/dL
Magnesium	1.5 mg/dL	1.4-1.9 mg/dL
Phosphorus	13.7 mg/dL	2.3-4.4 mg/dL
International Normalized Ratio (INR)	1.1	-
Prothrombin Time	13.6 seconds	11.5-14.4 seconds
Activated Partial Thromboplastin Time	28.7 seconds	24.4-36.2 seconds
Fibrinogen	454 mg/dL	235-490 mg/dL

The patient had progressive abdominal distension and pain, and on hospital day 3, he underwent a CT scan of the abdomen and pelvis without administered contrast, which showed new large-volume ascites, known polycystic kidney disease, and changes consistent with a previous Whipple procedure. There was no definitive radiologic evidence of metastatic disease, bowel obstruction, or other acute abnormality. He subsequently underwent a bedside paracentesis with 6 L of yellow-appearing ascites removed. Fluid analysis results are summarized in Table 2.

**Table 2:** Ascitic Fluid Analysis

Laboratory Test	Result
Appearance	Yellow, Cloudy
Total Nucleated Cells	307/cmm
Segmented Neutrophil	12%
Lymphocyte	29%
Monocyte	59%
Red Blood Cell Count	2000/cmm
Protein, Total, Fluid	4.4 g/dL
Albumin, Fluid	2.3 g/dL
Lactate Dehydrogenase, Fluid	133 U/L
Glucose, Fluid	124 mg/dL
Cholesterol, Fluid	78 mg/dL
Triglycerides	294 mg/dL
Creatinine	11.6 mg/dL
Amylase	30 U/L

An abdominal ultrasound with Doppler revealed a liver normal in size and appearance with patent hepatic vasculature. A transthoracic echocardiogram was performed and showed a left ventricular ejection fraction of 65%-70% with grade 1 diastolic dysfunction and an inferior vena cava diameter of 0.91 cm with greater than 50% collapse during respiration.

**Figure 1:**
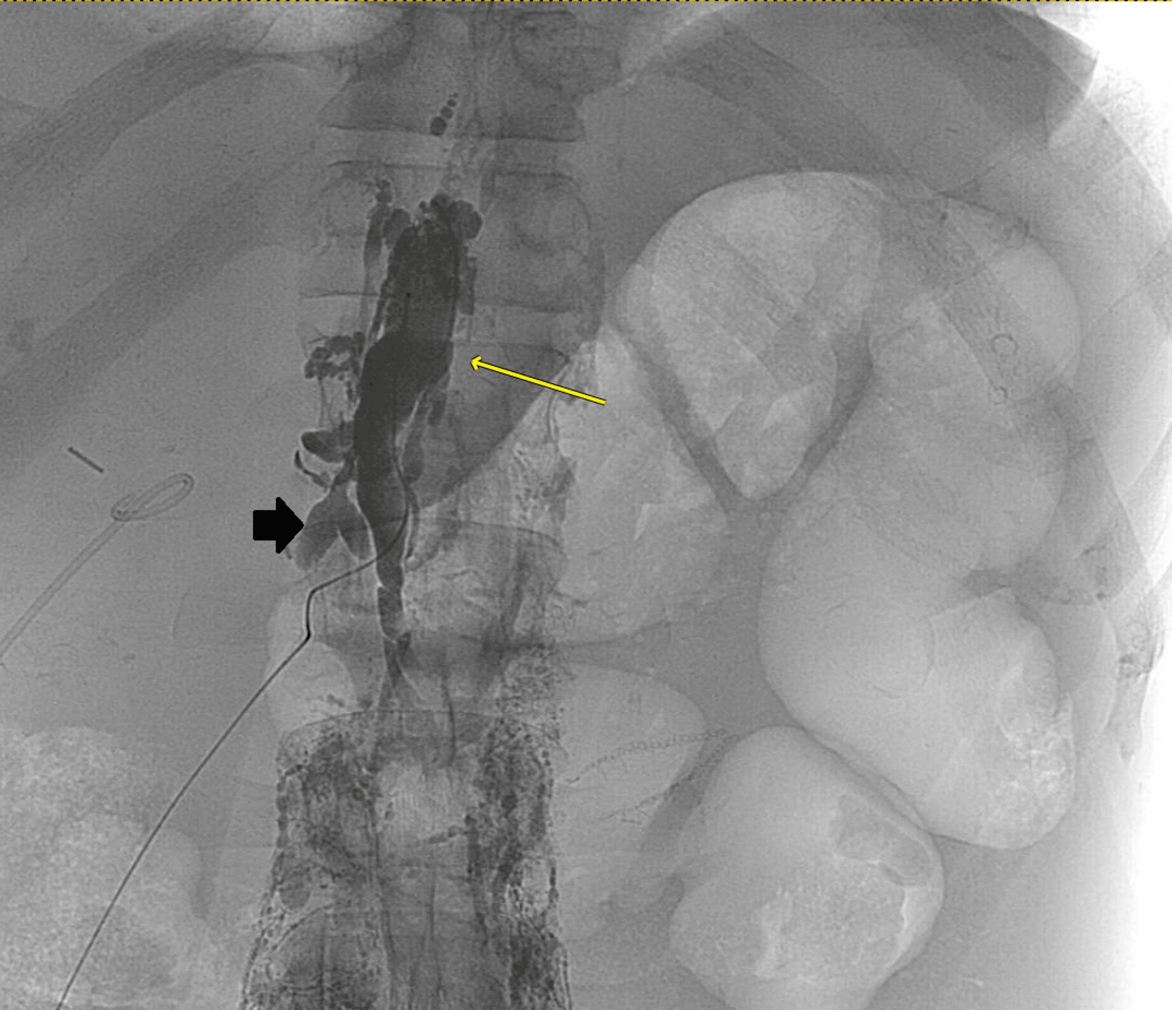
Lymphangiogram Lymphangiography with injected fluorescent dye opacifying the lymphatic system demonstrates a mega cisterna chyli (yellow arrow) and an adjacent irregularly shaped sac (black arrowhead) near the surgical clips, suggestive of lymphocele. No overt extravasation of dye into the peritoneal cavity is seen

Despite regularly scheduled sessions of intermittent hemodialysis with ultrafiltration and a fluid-restricted diet, the patient had a rapid re-accumulation of his ascites. He underwent three more paracenteses over the following two and a half weeks, with fluid removal ranging from 3 to 5.3 L. The serum-ascites albumin gradient (SAAG) on the fluid analysis of each paracentesis was <1.1 g/dL, and cytology on two separate samples was negative for malignant cells. Interventional radiology was consulted and attempted a transjugular liver biopsy with hepatic pressure measurements, but the procedure was aborted due to newly discovered venous thromboembolic occlusions of his bilateral internal jugular veins during the procedure. A percutaneous liver biopsy was performed with marked hemosiderosis on pathology but otherwise normal liver and biliary architecture. Given the rapid recurrent accumulation of ascitic fluid and concern for possible chylous ascites, the patient underwent lymphangiography (Figure 1). The patient subsequently underwent a successful embolization of the cisterna chyli, and 3 L of chylous ascites was concurrently removed. In the following months, the patient subsequently had a reduction in ascitic fluid accumulation but did still require occasional, less frequent paracenteses for therapeutic fluid removal.

## Discussion

This patient presented with rapid and progressive abdominal distension from chylous ascites after a Whipple procedure. In one series with 609 patients who underwent a Whipple procedure, 66 patients (11%) developed chylous ascites post-operatively, with a median time of onset of six days post-operatively, while in another series, 3% of 560 patients developed chylous ascites after a Whipple procedure [10,11]. While chylous ascites often develops early in the post-operative period, delayed presentation several weeks to months after can occur, representing an uncommon presentation of a less common condition.

While the patient had numerous potential etiologies for ascites, a systematic approach helped achieve the correct diagnosis. Common portal hypertensive causes, such as liver cirrhosis and heart failure, were felt to be unlikely given his lack of evidence of cirrhosis on examination and laboratory tests and a liver biopsy that was not consistent with cirrhosis. He also had a relatively normal transthoracic echocardiogram and repeatedly low SAAG on multiple analyses of his ascitic fluid. The attempted transjugular liver biopsy with pressure measurements would have helped further elucidate the presence or absence of elevated portal pressure but was unfortunately unable to be performed owing to the patient’s venous thromboembolic burden. Malignant ascites was also felt to be unlikely given the clean pathologic margins from his Whipple procedure, the lack of evidence of recurrent malignancy on multiple imaging studies, and the negative cytology on two separate samples of the ascitic fluid. The patient’s elevated ascitic triglyceride level above 200 mg/dL and SAAG of <1.1 g/dL suggested the diagnosis of chylous ascites, and the patient’s enlarged cisterna chyli on lymphangiography with subsequent reduction in ascites fluid accumulation after successful embolization further supported this diagnosis. While not definitive, it was suspected that trauma from the Whipple procedure caused a lymphocele formation. The delayed presentation was attributed in part to the timing of lymphocele formation and the subsequent slow leakage of chyle, with a contribution from missed hemodialysis sessions that likely allowed the intraperitoneal fluid to accumulate.

While this case identified an intervenable defect in the cisterna chyli that was amenable to embolization, medical management is often the first-line treatment in a majority of cases of a chyle leak. The aim of medical management is to reduce the flow of chyle to allow for the spontaneous closure of the fistula or the site of the leakage of lymphatic fluid [4]. This is achieved by fat-free or low-fat nutrition, which can be delivered by an oral diet and enteral feedings, or parenteral nutrition with high-protein contents and supplemented medium-chain triglycerides, which bypass the lymphatic system and are directly absorbed into the bloodstream. Consultation with a nutritionist is recommended [9]. Medical management also focuses on replacing any concomitant volume loss, correcting electrolyte derangements, supplementing fat-soluble vitamins, and closely monitoring the increased risk of infections. Somatostatin and octreotide are medications that can be used as adjunctive treatment, as they reduce chyle production and flow [4].

Consensus is lacking on when surgical intervention should be pursued for a chyle leak. In general, failed medical management and high chylous output are potential indications for surgical intervention. Chyle fluid loss of >1.5 L per day for >5-7 days despite medical management has been suggested as an indication to consider surgical intervention. Further, given the panoply of pathophysiologic mechanisms of chylous leaks, there is a wide range of potential surgical interventions, with some surgical series showing increased morbidity and mortality with open surgical interventions. Thus, caution is often exercised with risks and benefits carefully weighed prior to the consideration of surgical interventions [12].

Several minimally invasive techniques are now being used to treat this entity, including lipoidal lymphangiography; the embolization of the thoracic duct, lymphopseudoaneurysm, lymph node, and hepatic lymphatics; and/or peritoneovenous shunt [13,14]. The embolization of the cisterna chyli is a minimally invasive interventional radiology procedure that is frequently used in refractory cases of chylous ascites. It involves using lymphangiography to opacify the intraperitoneal lymphatic system, followed by embolization delivered by a transabdominal needle into the area where a suspected lymphatic lesion lies. In this case, it was used successfully to reduce the leakage of chylous fluid into the abdominal cavity and appears to be a potentially effective therapeutic strategy in select patients, particularly when a lymphatic leak can be identified. The reduction in the accumulation of ascitic fluid after intervention, but still ongoing need for intermittent paracenteses for fluid removal, likely reflects the contribution of ESRD to intra-abdominal volume accumulation, as well as potential refractory ongoing slow leakage of chylous fluid into the peritoneal cavity. This case of chylous ascites highlights a rare and serious complication of intra-abdominal surgery, as well as a successful embolization procedure to reduce the leakage of chyle.

## Conclusions

Chylous ascites is a rare cause of ascites that can portend a poor prognosis. Post-Whipple procedure as the cause of chylous ascites can have improved outcomes compared to some other causative etiologies of this condition, particularly with rapid identification and proper management. We present a case with multiple potential etiologies of ascites that ultimately was mainly attributed to lymph leakage after a Whipple procedure. Clinicians should consider this diagnosis in patients with milky-white ascites, triglyceride levels of >200 mg/dL in the ascites, rapidly accumulating ascites after abdominal surgery or procedures, and evidence of leakage from, or the enlargement of, the lymphatic system on imaging modalities, though they should recognize atypical presentations such as this case. Early recognition and a systematic diagnostic approach are critical to preventing complications and guiding appropriate therapeutic interventions.
